# Clinical characteristics and surgical history of Taiwanese patients with mucopolysaccharidosis type II: data from the hunter outcome survey (HOS)

**DOI:** 10.1186/s13023-018-0827-1

**Published:** 2018-06-04

**Authors:** Hsiang-Yu Lin, Chih-Kuang Chuang, Ming-Ren Chen, Shio Jean Lin, Pao Chin Chiu, Dau-Ming Niu, Fuu-Jen Tsai, Wuh-Liang Hwu, Yin-Hsiu Chien, Ju-Li Lin, Shuan-Pei Lin

**Affiliations:** 1 Department of Medicine, Mackay Medical College, New Taipei City, Taiwan; 2 Department of Pediatrics, Mackay Memorial Hospital, No. 92, Sec. 2, Chung-Shan North Road, Taipei, 10449 Taiwan; 3 Department of Medical Research, Mackay Memorial Hospital, Taipei, Taiwan; 4 Mackay Junior College of Medicine, Nursing and Management, Taipei, Taiwan; 5 Institute of Clinical Medicine, National Yang-Ming University, Taipei, Taiwan; 6 Department of Medical Research, China Medical University Hospital, China Medical University, Taichung, Taiwan; 7 Medical College, Fu-Jen Catholic University, Taipei, Taiwan; 8 Institute of Biotechnology, National Taipei University of Technology, Taipei, Taiwan; 9 Department of Pediatrics, Chi Mei Medical Center, Tainan, Taiwan; 10 Department of Pediatrics, National Yang-Ming University, Taipei, Taiwan; 11 Department of Pediatrics, Kaohsiung Veterans General Hospital, Kaohsiung, Taiwan; 12 Department of Pediatrics, Taipei Veterans General Hospital, Taipei, Taiwan; 13 Department of Pediatrics, China Medical University Hospital, Taichung, Taiwan; 14 Department of Pediatrics, National Taiwan University Hospital, Taipei, Taiwan; 15 Division of Genetics and Endocrinology, Department of Pediatrics, Chang Gung University College of Medicine and Chang Gung Children’s and Memorial Hospital, Taoyuan, Taiwan; 16 Department of Infant and Child Care, National Taipei University of Nursing and Health Sciences, Taipei, Taiwan

**Keywords:** Causes of death, Diagnosis, Hernia repair, Hunter syndrome, Lysosomal storage disease, Surgery, Survival, Symptoms

## Abstract

**Background:**

Mucopolysaccharidosis type II (MPS II) is the most frequently occurring MPS in Taiwan, with an incidence of 2.05 per 100,000 live male births, but little is known about clinical characteristics and surgical history in Taiwanese patients.

**Methods:**

Medical history, demographics, signs and symptoms, and surgical history were analysed in all patients from Taiwanese centres in the Hunter Outcome Survey (HOS; NCT 03292887), a global, multicentre registry that collects real-world data on patients with MPS II.

**Results:**

As of January 2016, 61 male Taiwanese patients were enrolled; 49% (24/49) had received at least one infusion of idursulfase. Median (10th, 90th percentiles) ages at signs and symptom onset and at diagnosis were 2.5 (0.2, 5.5) years (*n* = 55) and 3.5 (1.2, 11.9) years (*n* = 56), respectively. Hernia, facial features consistent with MPS II and claw hands were the earliest presenting signs and symptoms (median ages of 3.2 [0.4, 12.0] years, 4.3 [1.1, 12.0] years and 4.7 [2.5, 12.2] years [*n* = 45, 53 and 50], respectively). More than 75% of patients had undergone a surgical procedure, most commonly hernia repair (57% of patients). Median age at first surgery for hernia repair was 4.2 (0.5, 9.8) years (*n* = 35). Almost one-third (31.1%) of patients had at least one surgical procedure before diagnosis, and of the 20 procedures before diagnosis, 16 were hernia repair.

**Conclusions:**

This information from patients in HOS highlights the importance of both medical and surgical history in diagnosing MPS II in Taiwanese patients.

**Electronic supplementary material:**

The online version of this article (10.1186/s13023-018-0827-1) contains supplementary material, which is available to authorized users.

## Background

Mucopolysaccharidosis type II (MPS II; Hunter syndrome; OMIM 309900) is a rare, X-linked, life-limiting disease caused by deficient activity of the lysosomal enzyme iduronate-2-sulfatase (I2S; EC 3.1.6.13) [1]. I2S deficiency causes progressive accumulation of glycosaminoglycans (GAGs) in tissues and organs, contributing to the progressive multisystem clinical signs and symptoms [1]. The disease has an estimated global incidence of 0.6–1.3 per 100,000 live male births [2, 3]. In Taiwan, MPS II is the most frequently occurring MPS (incidence: 2.05 per 100,000 live male births) [4].

Patients usually present with signs and symptoms of MPS II between 2 and 4 years of age, but diagnosis is typically not confirmed until several years later [5, 6]. Surgical intervention at a young age is often required and frequently occurs before formal diagnosis of MPS II [5]. Patterns of certain types of surgery (i.e. hernia repair, ear tube insertion, adenoidectomy, tonsillectomy and carpal tunnel release) are characteristic of MPS II [5], and general awareness of typical surgical patterns may aid early diagnosis and timely initiation of disease management [5, 7, 8].

For clinical purposes, patients are generally considered to fall into one of two categories [1, 9–11]. Individuals with cognitive impairment tend to have severe somatic manifestations and early onset of disease and typically survive to the second decade of life; in those without cognitive impairment, somatic involvement can range from severe with early onset to much less severe with later onset, and less severely affected patients may survive to their fifth or sixth decade [1, 12, 13].

Specific treatment for patients with MPS II is available in the form of enzyme replacement therapy (ERT) with recombinant I2S (idursulfase, Elaprase®; Shire, Lexington, MA, USA), administered intravenously (0.5 mg/kg of body weight once weekly). Idursulfase received marketing authorization in Taiwan in August 2012. Clinical trials have provided evidence that ERT with idursulfase improves clinical parameters [14–16].

The Hunter Outcome Survey (HOS) is a large, global, multicentre, observational registry (Shire, Lexington, MA, USA; NCT 03292887) that was initiated in 2005. The registry collects long-term data on patients with MPS II and the efficacy and safety of ERT with idursulfase [5, 6, 12, 16–32]. Data from patients in Taiwan have been entered in the registry since 2012. Retrospective chart reviews have revealed insights into cardiovascular abnormalities, pulmonary function impairments and causes of death in Taiwanese patients with MPS II; however, patient numbers were low and there have been no published analyses of surgical history [33–35]. This analysis is the first to investigate a broad range of clinical characteristics and surgical history in a large population of Taiwanese patients with MPS II who were enrolled in HOS as of January 2016.

## Methods

### Registry design

HOS is designed to collect a range of disease- and treatment-related data from individuals with MPS II during routine patient assessments (both prospectively and retrospectively) [6]. Individuals with MPS II who are not treated and those who are receiving treatment with idursulfase are eligible for enrolment in the registry (patients receiving treatment with an ERT product other than Elaprase are not eligible for inclusion). Institutional Review Board/Ethics Committee approval is obtained for all participating centres. Written informed consent is obtained from each patient, or their parents or legal representative. For patients who died before HOS entry, consent is obtained from patients’ families. All patient information is managed in accordance with national data protection standards.

### Patient population

As of January 2016, there were 1096 patients enrolled in HOS from 124 centres in 29 countries. Patients from Taiwan who were alive at registry entry were followed prospectively (prospective patients) and had data collected at enrolment and at subsequent routine clinical visits at participating centres. In addition, data from patients who died before enrolment (retrospective patients) were also entered into the database. All patient information is managed in accordance with local country data protection standards.

### Data collection and analysis

For all patients for whom data were available, this study analysed data relating to selected key clinical parameters, including medical history and demographic information, and occurrence of and age at onset of signs and symptoms. For patients receiving ERT with idursulfase, age at treatment initiation and length of time on ERT were analysed. The presence or absence of cognitive impairment was determined by the assessing healthcare professional on the basis of the answer to the following question: ‘Cognitive impairment? Yes/No’ for the period from birth to registry entry and at subsequent visits (i.e. at any time). Assessment of cognitive impairment was based on clinical impression and/or the results of standardized testing.

Surgical procedures recorded in the database were analysed. ‘Other’ is a category in which surgical procedures not covered by the main database fields may be recorded using free text. In some cases, a surgical procedure recorded using free text was considered to fall under a main database category; in these instances, surgical procedures originally listed as ‘Other’ were reclassified into the appropriate main category, and the reclassification was verified by the HOS Biostatistician and the HOS Medical Monitor.

Data for causes of death recorded were also analysed.

This study presents the data for the overall patient population and for prospective and retrospective patients.

### Statistical methods

Descriptive statistics were used to summarize demographic data, with mean values (standard deviation) and median values (10th and 90th percentiles [P10, P90]) presented. For analyses of signs and symptoms and surgical history, the median values alone are given. Survival times were estimated using the Kaplan–Meier method for all patients, as well as for patients with cognitive impairment and for those without. Any patients not known to have died were assumed to be alive and were censored at their most recent recorded visit.

## Results

### Patient population

In total, 61 Taiwanese patients with MPS II were included in the analysis, all of whom were male. Of these individuals, 44 were prospective patients and 17 were retrospective patients.

The demographics and baseline characteristics of the patients in this analysis are shown in Table 1. In the overall population (both prospective and retrospective patients), the median age (P10, P90) at onset of signs and symptoms was 2.5 (0.2, 5.5) years (*n* = 55), and the median age at diagnosis was 3.5 (1.2, 11.9) years (*n* = 56). The median age at diagnosis was slightly lower in patients with a positive family history of MPS II (56.0%; 28/50) than in those without: 3.3 (1.0, 15.3) years and 3.8 (1.9, 6.3) years, respectively. This pattern was also seen in prospective patients; however, retrospective patients with a family history of MPS II had a later age at diagnosis than those without (Additional file 1: Table S1). More than 70% (40/56) of the patients had cognitive impairment, and almost half (49.0%; 24/49) had received at least one infusion of idursulfase.

**Table 1:** Demographics and baseline characteristics of patients included in this analysis

Characteristic	Overall population (N = 61)	Prospective patients (*n* = 44)	Retrospective patients (*n* = 17)
Age at HOS entry			
Number of patients with data available	61	44	17
Mean (SD), years	13.5 (7.0)	13.6 (7.8)	13.4 (4.5)
Median (P10, P90), years	13.2 (5.1, 22.4)	13.4 (4.6, 23.2)	13.0 (9.5, 19.4)
Age at onset of signs and symptoms			
Number of patients with data available	55	38	17
Mean (SD), years	2.9 (3.2)	3.1 (3.3)	2.6 (2.8)
Median (P10, P90), years	2.5 (0.2, 5.5)	2.5 (0.3, 5.8)	1.7 (0.1, 5.5)
Age at diagnosis			
Number of patients with data available	56	39	17
Mean (SD), years	5.3 (5.4)	5.6 (6.0)	4.6 (3.6)
Median (P10, P90), years	3.5 (1.2, 11.9)	3.4 (1.2, 13.3)	3.6 (0.1, 10.8)
Ethnicity			
Asian, n/N (%)	59/60 (98.3)	43/44 (97.7)	16/16 (100)
Other, n/N (%)	1/60 (1.7)	1/44 (2.3)	0/16 (0)
Family history of MPS II, yes, n/N (%)	28/50 (56.0)	18/35 (51.4)	10/15 (66.7)
Sex, male, n/N (%)	61/61 (100)	44/44 (100)	17/17 (100)
Treated with idursulfase at any time, yes, n/N (%)	24/49 (49.0)	24/38 (63.2)	0/11 (0)
Age at idursulfase treatment start			
Number of patients with data available	24	24	N/A
Mean (SD), years	13.4 (9.2)	13.4 (9.2)	
Median (P10, P90), years	12.5 (4.5, 24.9)	12.5 (4.5, 24.9)	
Length of time on idursulfase treatment			
Number of patients with data available	24	24	N/A
Mean (SD), months	43.8 (27.0)	43.8 (27.0)	
Median (P10, P90), months	41.6 (9.3, 79.3)	41.6 (9.3, 79.3)	
Cognitive impairment at any time, yes, n/N (%)	40/56 (71.4)	24/39 (61.5)	16/17 (94.1)
Age at last visit in HOS			
Number of patients with data available	44	44	
Mean (SD), years	16.9 (8.2)	16.9 (8.2)	N/A
Median (P10, P90), years	17.0 (7.3, 25.0)	17.0 (7.3, 25.0)	
Deceased, yes, n/N (%)	25/61 (41.0)	8/44 (18.2)	17/17 (100)
Age at death			
Number of patients with data available	25	8	17
Mean (SD), years	14.4 (4.6)	16.4 (4.3)	13.4 (4.5)
Median (P10, P90), years	13.4 (9.5, 19.4)	16.7 (9.8, 24.1)	13.0 (9.5, 19.4)

The overall population contains both prospective and retrospective patients. Patient numbers are fewer than 61 for some characteristics due to data not being entered into HOS

*HOS* Hunter Outcome Survey, *MPS II* mucopolysaccharidosis type II, *N/A* not applicable, *P10, P90* 10th and 90th percentiles, *SD* standard deviation

The majority of demographics and baseline characteristics investigated were similar for prospective and retrospective patients. Of note, a larger proportion of retrospective patients than prospective patients was reported to have cognitive impairment (94.1 and 61.5%, respectively).

### Signs and symptoms of MPS II

**Fig. 1:**
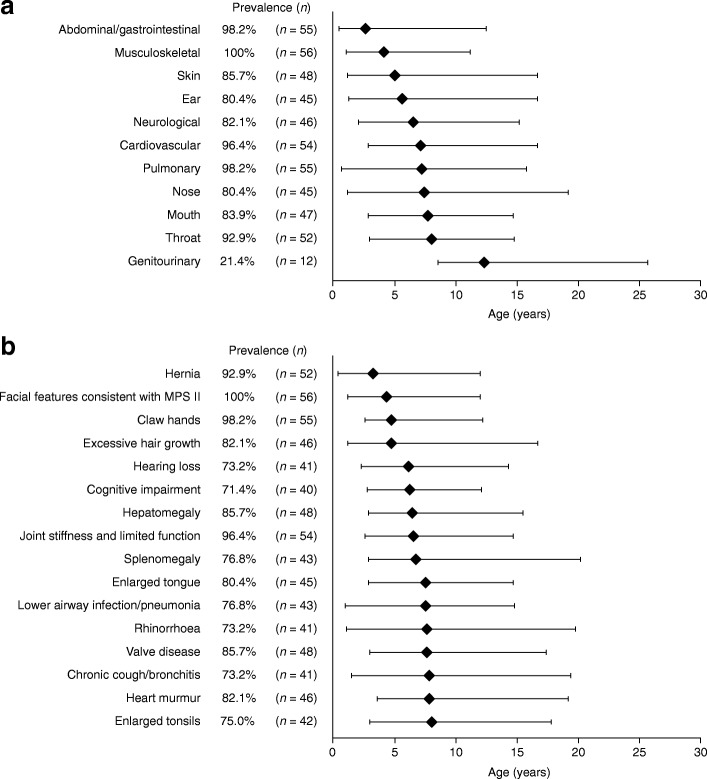
Disease manifestations and signs and symptoms in the overall population. Prevalence and median age at onset of (**a**) organ system involvement (*n* = 56) and (**b**) signs and symptoms present in more than 70% of patients in the overall population. The overall population contains both prospective and retrospective patients. Diamonds represent the median age of onset (years). Error bars indicate the 10th and 90th percentiles. Patient numbers for median age at onset of organ system involvement (*n* = 11–53) and signs and symptoms (*n* = 33–53) may differ from those for prevalence due to data available in the Hunter Outcome Survey. MPS II, mucopolysaccharidosis type II

The musculoskeletal system was affected in all patients in the overall population with available data (100%; 56/56); involvement of the abdominal/gastrointestinal system and the pulmonary system was reported in 98.2% of patients (55/56 for both) (Fig. 1a). The signs and symptoms that were reported in at least 70% of patients are presented in Fig. 1b. The most prevalent signs and symptoms were facial features consistent with MPS II (100%; 56/56), claw hands (98.2%; 55/56), joint stiffness and limited function (96.4%; 54/56) and hernia (92.9%; 52/56; inguinal: 75.0%; 42/56; umbilical: 75.0%; 42/56). The earliest presenting characteristics of MPS II included hernia, facial features consistent with MPS II and claw hands, which had median ages (P10, P90) at onset of 3.2 (0.4, 12.0), 4.3 (1.1, 12.0) and 4.7 (2.5, 12.2) years, respectively (*n* = 45, 53 and 50, respectively).

The majority of signs and symptoms had a similar prevalence in, or were less prevalent in, retrospective patients compared with prospective patients; however, hernia, cognitive impairment, lower airway infection/pneumonia and dyspnoea were reported more frequently in retrospective patients (Additional file 1: Figure S1). The median age at onset of the most common signs and symptoms (those reported in 70% or more of individuals) in retrospective patients was similar to or less than that reported for prospective patients.

### Surgical history

**Fig. 2:**
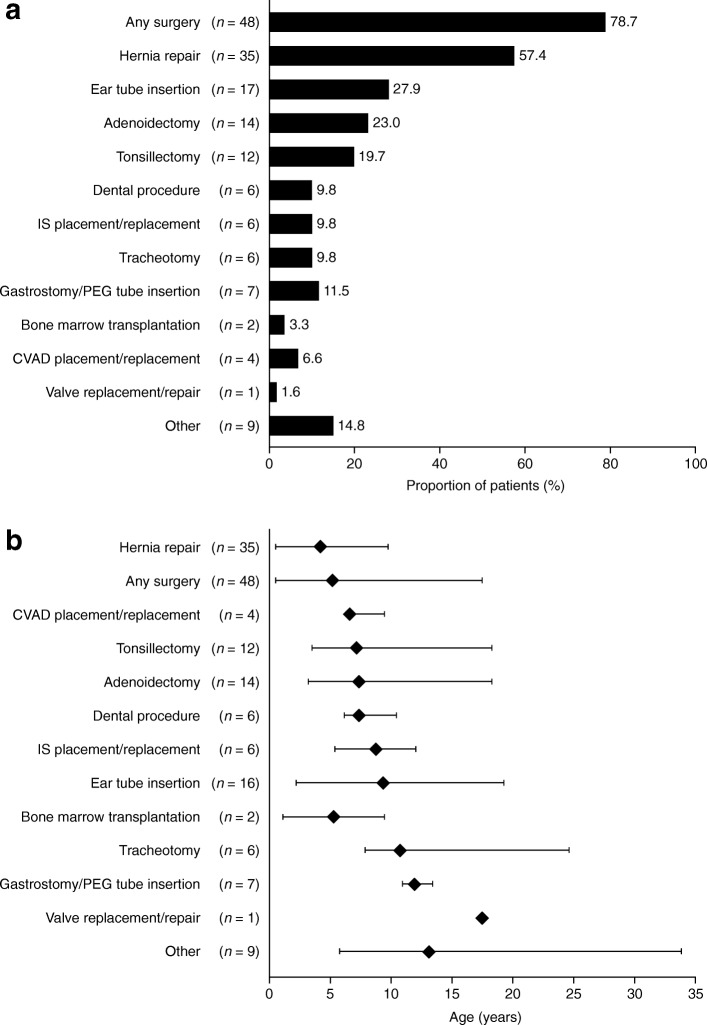
Surgical procedures in the overall population. (**a**) Percentage of patients in the overall population undergoing surgical procedures at any time (*N* = 61). (b) Median age at first surgical procedure for surgeries performed in the overall population. The overall population contains both prospective and retrospective patients. In part (**b**), diamonds indicate the median age of onset (years) and bars indicate the 10th and 90th percentiles. ‘Other’ is a category in the database in which surgical procedures not covered by the main database fields may be recorded using free text; this category included, but was not limited to, heel cord lengthening and pacemaker implantation. In some cases, a surgical procedure recorded using free text was considered to fall under a main database category; in these instances, surgical procedures originally listed as ‘Other’ were reclassified into the appropriate main category and the reclassification was verified by the HOS Biostatistician and the HOS Medical Monitor. CVAD, central venous access device; IS, intracranial shunt; PEG, percutaneous endoscopic gastrostomy

Figure 2 shows the frequency and age at surgery for all surgical interventions recorded in the Taiwanese population in HOS. In the overall population, 78.7% (48/61) of patients had undergone at least one surgical procedure at any time (Fig. 2a). The most common surgical procedures were hernia repair (57.4%; 35/61), ear tube insertion (27.9%; 17/61), adenoidectomy (23.0%; 14/61) and tonsillectomy (19.7%; 12/61). Surgical procedures classed as ‘Other’ were reported for 14.8% of the patients (9/61) (Fig. 2a). Carpal tunnel release was not reported in this population. The earliest type of surgery performed was hernia repair, with a median age at surgery of 4.2 (0.5, 9.8) years (*n* = 35) (Fig. 2b).

Almost one-third of patients (31.1%; 19/61) underwent surgery before receiving a confirmed diagnosis of MPS II. In total, 20 procedures were performed in these 19 patients; these comprised 16 hernia repairs (at a median age of 0.9 [0.4, 4.7] years), two ear tube insertions (median age 2.2 [2.1, 2.2] years) and two surgeries categorized as ‘Other’ (median age 12.1 [5.8, 18.5] years). The proportion of these patients with a positive family history of MPS II was similar to that of individuals with a negative family history (42.1 and 47.4%, respectively).

More retrospective patients than prospective patients underwent surgery at any time (88.2 and 75.0%, respectively) (Additional file 1: Figure S2). In both groups, hernia repair was the most common surgical procedure (this occurred in a similar proportion of patients in each group) and it had the earliest median age at first surgery. With the exception of adenoidectomy and central venous access device placement/replacement, the pattern of prevalence of the remaining surgeries varied between prospective and retrospective patients. Age at first ear tube insertion occurred at 11.3 (2.1, 11.7) years in retrospective patients (*n* = 3) and 7.6 (2.5, 19.4) years in prospective patients (*n* = 13). Age at first tracheotomy was 9.5 (7.9, 13.3) years in retrospective patients (*n* = 4) and 18.5 (12.1, 24.8) years in prospective patients (*n* = 2).

### Survival

In the overall population, 41.0% (25/61) of the patients had died at the time of this analysis. The leading causes of death in the overall population were respiratory failure (44.0%; 11/25) and cardiac arrest/failure (24.0%; 6/25), followed by pneumonia (12.0%; 3/25), infection (sepsis) (4.0%; 1/25) and accident (4.0%; 1/25); the causes of three deaths (12.0%) were unknown.

**Fig. 3:**
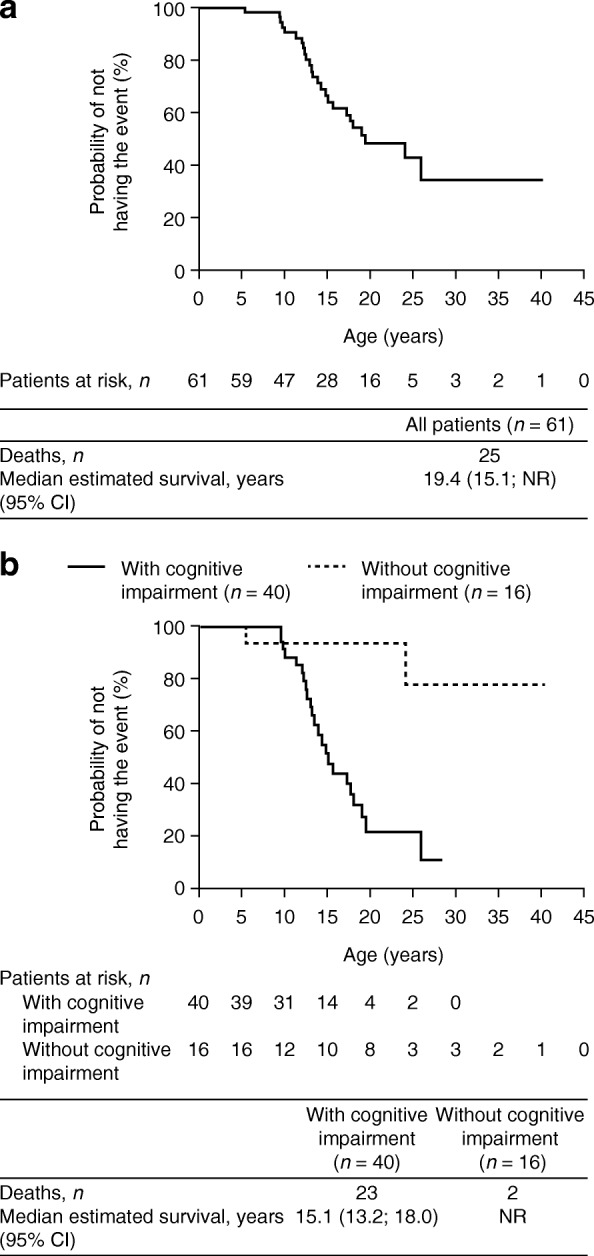
Survival in the overall population and according to cognitive impairment. Kaplan–Meier survival analysis for (**a**) all patients in the overall population (N = 61) and (**b**) patients in the overall population with and without cognitive impairment (*n* = 40 and *n* = 16, respectively). The overall population contains both prospective and retrospective patients. CI, confidence interval; NR, not reached

A larger proportion of prospective patients than retrospective patients died due to cardiac arrest/failure (37.5% vs 17.6%, respectively) (Additional file 1: Figure S3). Respiratory failure was reported in a larger proportion of retrospective patients than prospective patients (52.9% vs 25.0%, respectively). Kaplan–Meier survival curves of all patients (*N* = 61) and according to cognitive impairment status (*n* = 56) are shown in Fig. 3a and b, respectively. The median survival estimate (95% confidence interval) for all patients was 19.4 (15.1, not reached) years. Estimated median survival for individuals in the overall population with cognitive impairment was 15.1 (13.2, 18.0) years; 23 of the 40 patients with cognitive impairment had died. Only two deaths were reported in the 16 patients without cognitive impairment, and median survival could not be estimated.

Kaplan–Meier survival curves for prospective and retrospective patients, and also according to cognitive impairment status, are shown in Additional file 1: Figure S4. Eight of the 44 prospective patients had died: seven of the 24 prospective patients with cognitive impairment and one of the 15 prospective patients without cognitive impairment. Of the 17 retrospective patients in this analysis, 16 were reported to have had cognitive impairment. Patient numbers were generally low and in some cases were too low for survival probabilities to be estimated.

## Discussion

This is the first analysis of a broad range of clinical manifestations and surgical histories of a large population of Taiwanese patients with MPS II. Clinical manifestations attributed to musculoskeletal, abdominal/gastrointestinal and pulmonary systems were reported for the majority of patients. More than three-quarters of the patients had undergone a surgical procedure, with hernia repair being both the most common and the earliest surgery performed. Almost one-third of patients underwent surgery before receiving a diagnosis of MPS II; most of the operations performed before diagnosis were hernia repair.

In Taiwan, MPS II is the most frequently occurring MPS (incidence: 2.05 per 100,000 live male births) [4]. Median ages at signs and symptoms onset and diagnosis in the Taiwanese population in this analysis were similar to those reported previously in the global HOS population (2.5 years vs 1.5 years and 3.5 years vs 3.5 years, respectively) [6]. Consistent with other findings, facial features consistent with MPS II was the most common clinical manifestation of MPS II [6]. Hernia was more common in the Taiwanese population than reported previously in the global HOS population (93% vs 78%, respectively) but occurred at an older age (3.2 years vs 1.3 years) [6]. Other signs and symptoms more common in this study population than previously reported in the global HOS population were claw hands (98.2% vs 53.2% [prospective patients only] [21]) and valve disease (85.7% vs 57% [6] and 63% [prospective patients only] [23]); however, ages at onset were similar. The finding that musculoskeletal and abdominal/gastrointestinal systems were among the most commonly affected organ systems is broadly similar to that previously reported globally in HOS; however, pulmonary, cardiovascular and throat signs and symptoms were slightly more common in the Taiwanese population [6]. Age at onset of signs and symptoms tended to be older in Taiwanese patients for joint stiffness and limited function, enlarged tonsils, enlarged tongue and otitis media compared with previous reports for the global HOS population [6, 21, 24]. Further work is required to investigate the possible reasons for these findings.

The current analysis is the first to describe surgical history in Taiwanese patients with MPS II. Similar to findings in other populations of patients in HOS, more than three-quarters of the Taiwanese patients in this analysis had undergone a surgical procedure, and more than half underwent hernia repair [5]. However, some surgeries were reported less frequently in Taiwanese patients than in the global HOS population: ear tube insertion (27.9% vs 51.4%), adenoidectomy (23.0% vs 49.5%) and tonsillectomy (19.7% vs 35.5.%) [5]. Interestingly, carpal tunnel release was not reported in any patient in this population, despite being commonly reported in a global analysis of HOS data [5]. It may be that clinicians and caregivers in Taiwan, as compared with those in the rest of the world, have different levels of awareness of the need for surgery in MPS II that could explain these differences in frequency of surgical procedures in Taiwanese patients.

Age at first surgery tended to be older in Taiwanese patients than in the global HOS population (prospective patients: 5.1 years vs 2.6 years) [5]. However, a considerable proportion of patients in this analysis (31.1%) had undergone a surgical procedure before diagnosis. Even though this finding is lower than that reported for the global HOS population (56.8%) [5], it is in line with what is known in MPS II: patients may first present to a general paediatrician [8], and many individuals will be undiagnosed when they are first referred to a surgeon [5]. Also of note are the findings in Taiwanese patients that hernia repair is both the most common and the earliest surgery, and is also the most common surgery before diagnosis. This is in concordance with hernia being the first sign and symptom noted in this patient population. Therefore, it is important that clinicians in Taiwan treating paediatric patients with high rates of surgical procedures, particularly surgery for hernia repair, consider MPS II as a potential diagnosis. Early diagnosis permits early initiation of ERT, which may be beneficial, particularly before organ damage becomes irreversible [13, 36].

In agreement with data from the global HOS population and a study of retrospective patients in Taiwan [1, 12, 13, 31, 35], the leading causes of death reported in this analysis were respiratory failure and cardiac arrest/failure. A high prevalence of pulmonary function impairment was previously reported in patients with MPSs in Taiwan: 91% were found to have small airway disease, 48% had restrictive lung disease and 9% had obstructive lung disease [34]. Cardiovascular abnormalities have also been found to be common in Taiwanese patients with MPSs; over 80% of those with MPS II were reported to have deformed aortic or mitral valves [33]. The recent report on survival and causes of death in prospective patients in the global HOS population highlights that a single cause of death is unlikely to reflect the full clinical situation in a patient with MPS II, but that ERT with idursulfase is nonetheless associated with increased survival [31]. Previous reports have suggested a shorter life expectancy in patients with cognitive impairment than in those without cognitive impairment [6, 12, 31, 35]. Although this trend was seen in this analysis, the patient numbers are too small to draw this conclusion for this population or to investigate the effects of ERT.

It is important to consider the limitations of this study. The analysis population included both prospective and retrospective patients. Retrospective patients, who died before enrolment, are likely to have received care before modern standards and approaches were implemented, and in some cases only limited data are available for these individuals. This could, in part, explain the differences between prospective and retrospective patients in terms of the patterns and age at onset of the signs and symptoms investigated. The fact that the analysis population contained both patients who were untreated and those who were treated with idursulfase must also be taken into consideration; a greater number of patients and longer follow-up would be needed to investigate treatment effects.

As with any registry, patient enrolment and data entry in HOS are at the discretion of the participating centres. Information was obtained during routine visits and according to local practice; the frequency and the types of investigation carried out may vary between centres, and, as with any registry, there are limitations in the depth and nature of information that it is possible to collect [29]. In addition, the presence of cognitive impairment was determined using a ‘yes/no’ question from the database, the answer to which may not have been decided using standardized cognitive tests but may instead have been based on subjective clinical impression.

Despite these limitations, registry data are a valuable source of information about patients with MPS II in the real-world clinical setting and enable longer follow-up in a larger population than is usually possible in clinical trials. This is the first and largest analysis of clinical manifestations and surgical histories of Taiwanese patients from the HOS registry and may be considered to be a fair representation of the overall population of patients with MPS II in Taiwan. By continuing to record and analyse clinical and laboratory data from a wide variety of patients, we will enhance our understanding of MPS II in Taiwan. Further work is needed to determine whether potential differences between the Taiwanese and global populations of patients with MPS II are clinically significant.

## Conclusions

This analysis found that, in Taiwanese patients with MPS II, hernia, facial features consistent with the disease and claw hands were the earliest presenting signs and symptoms. Many of the patients underwent a surgical procedure, particularly hernia repair, at a young age and often before diagnosis. Based on these data, characterization of this patient group with MPS II may lead to early diagnosis and optimal management of the disease. An understanding of the disease progression of MPS II and characterization of both the clinical impairments and the surgical history in Taiwanese patients could be used to develop quality-of-care strategies as well as provide guidance on clinical trial outcomes and diagnostic processes for these patients.

## Additional file

Additional file 1: **Table S1.** Age at diagnosis by family history of MPS II in prospective and retrospective patients. **Figure S1.** Disease manifestations and signs and symptoms in prospective and retrospective patients. **Figure S2.** Surgical procedures in prospective and retrospective patients. **Figure S3.** Causes of death in prospective and retrospective patients. **Figure S4.** Survival in prospective and retrospective patients and according to cognitive impairment. (DOCX 292 kb)

## Abbreviations

ERT: enzyme replacement therapy
GAG: glycosaminoglycan
HOS: Hunter Outcome Survey
I2S: iduronate-2-sulfatase
MPS II: mucopolysaccharidosis type II
