# Supplementary material for: Clinical characteristics and surgical history of Taiwanese patients with mucopolysaccharidosis type II: data from the hunter outcome survey (HOS)
Source: Orphanet J Rare Dis. 2018 Jun 4;13:89. doi: 10.1186/s13023-018-0827-1 (PMC5987665; doi:10.1186/s13023-018-0827-1)

**Additional file**

**Clinical characteristics and surgical history of Taiwanese patients with mucopolysaccharidosis type II: data from the Hunter Outcome Survey (HOS)**

Hsiang-Yu Lin^1–5^, Chih-Kuang Chuang^3,6,7^, Ming-Ren Chen^1,2,4^, Shio Jean Lin^8^, Pao Chin Chiu^9,10^, Dau-Ming Niu^5,11^, Fuu-Jen Tsai^12^, Wuh-Liang Hwu^13^, Yin-Hsiu Chien^13^, Ju-Li Lin^14^ and Shuan-Pei Lin^1–4,15^

**Table S1** Age at diagnosis by family history of MPS II in prospective and retrospective patients

|  | **Prospective patients  (n = 44)** | | **Retrospective patients  (n = 17)** | |
| --- | --- | --- | --- | --- |
| **Family history of MPS II?** | **Yes** | **No** | **Yes** | **No** |
| Age at diagnosis  Number of patients with data available  Mean (SD), years  Median (P10, P90), years | 18  6.7 (8.3)  3.0 (1.0, 18.8) | 17  4.6 (2.8)  4.1 (1.9, 10.1) | 10  5.3 (3.9)  4.3 (0.7, 11.5) | 5  2.9 (1.8)  3.0 (0.1, 5.0) |

Patient numbers for prospective and retrospective patients are less than 44 and 17, respectively, due to data available in HOS.

HOS, Hunter Outcome Survey; MPS II, mucopolysaccharidosis type II; P10, P90, 10th and 90th percentiles.

**Fig. S1** Disease manifestations and signs and symptoms in prospective and retrospective patients. Prevalence and median age at onset of (a) organ system involvement in prospective and retrospective patients, and (b) signs and symptoms present in more than 70% of these patients. Diamonds indicate the median age of onset (years). Error bars indicate the 10th and 90th percentiles. Patient numbers for median age at onset of organ system involvement (prospective n = 10–36, retrospective n = 1–17) and signs and symptoms (prospective n = 11–36, retrospective n = 9–17) may differ from those for prevalence according to data available in the Hunter Outcome Survey. MPS II, mucopolysaccharidosis type II.


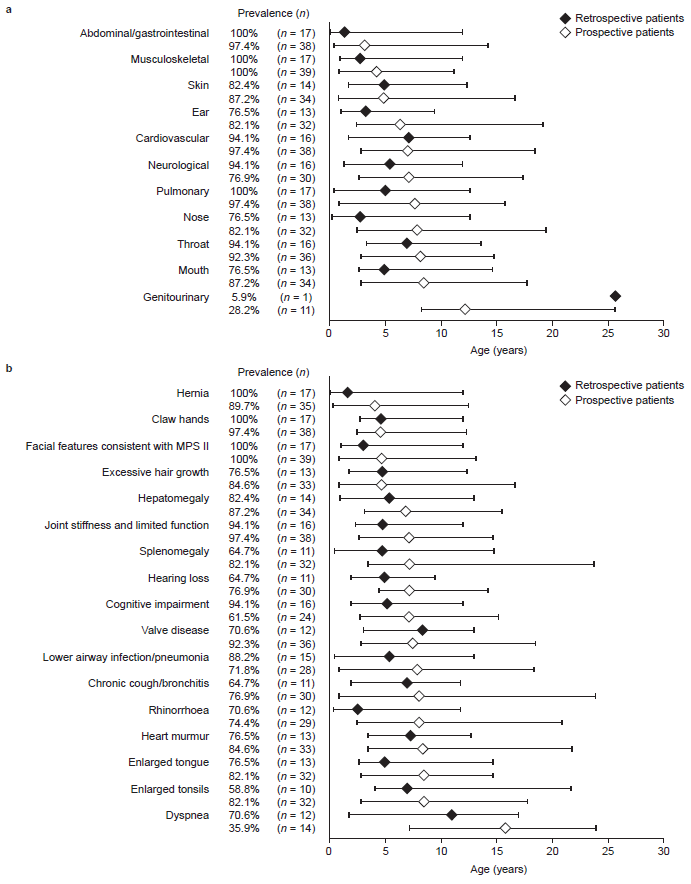


**Fig. S2** Surgical procedures in prospective and retrospective patients. (a) Percentage of patients undergoing surgical procedures at any time in prospective and retrospective patients. (b) Median age at first surgical procedure for surgeries performed in these patients. ‘Other’ is a category in the database in which surgical procedures not covered by the main database fields may be recorded using free text. In some cases, a surgical procedure recorded using free text was considered to fall under a main database category; in these instances, surgical procedures originally listed as ‘'Other’ were reclassified into the appropriate main category and the reclassification was verified by the HOS Biostatistician and the HOS Medical Monitor. CVAD, central venous access device; HOS, Hunter Outcome Survey; IS, intracranial shunt; PEG, percutaneous endoscopic gastrostomy.


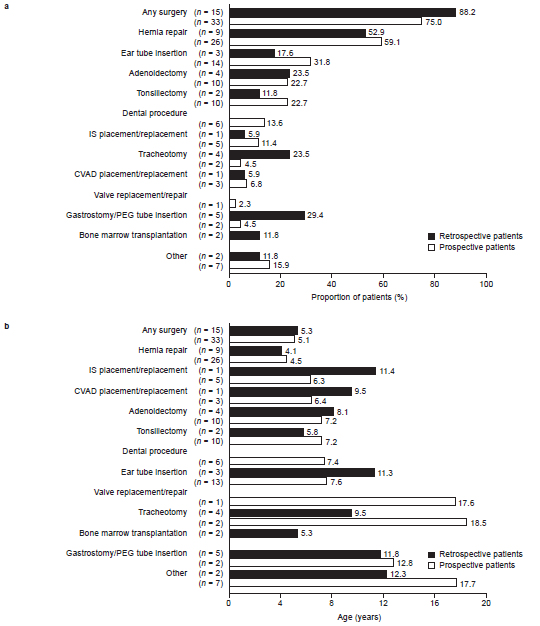


**Fig. S3** Causes of death in prospective and retrospective patients. Patient numbers are given above the bars.


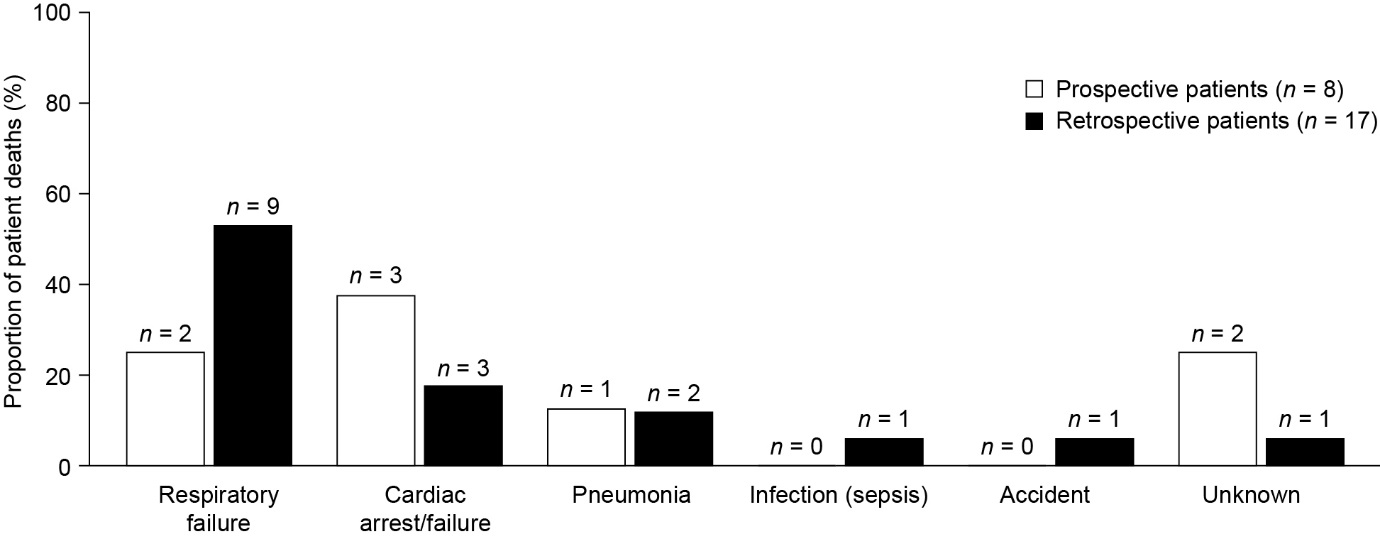


**Fig. S4** Survival in prospective and retrospective patients and according to cognitive impairment. Kaplan–Meier survival analyses for (a) prospective and retrospective patients, and (b) prospective and retrospective patients according to cognitive impairment status. CI, confidence interval; NR, not reached.


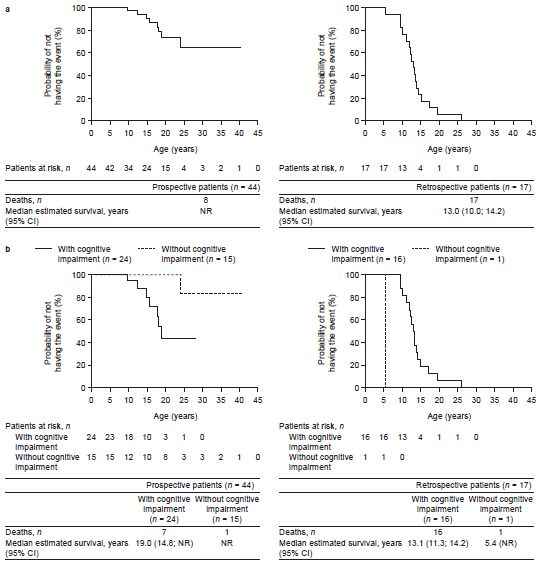

Supplement: Supplementary file 1 — Table S1. Age at diagnosis by family history of MPS II in prospective and retrospective patients. Figure S1. Disease manifestations and signs and symptoms in prospective and retrospective patients. Figure S2. Surgical procedures in prospective and retrospective patients. Figure S3. Causes of death in prospective and retrospective patients. Figure S4. Survival in prospective and retrospective patients and according to cognitive impairment. (DOCX 292 kb) [file 13023_2018_827_MOESM1_ESM.docx]
